# Supplementary material for: Bayesian multivariate reanalysis of large genetic studies identifies many new associations
Source: PLoS Genet. 2019 Oct 9;15(10):e1008431. doi: 10.1371/journal.pgen.1008431 (PMC6802844; doi:10.1371/journal.pgen.1008431)
Supplement: S1 Fig — Shown here is a Directed Acyclic Graphical (DAG) model of our multivariate categories in the context of our vector of phenotypes Y (e.g. Y = {YU, YD, YI}) and their connections with the variant of interest g. The relationships described in-text can be seen here. YU, our unassociated phenotypes, have no connection with g. YD, our directly associated phenotypes, have a direct connection with g. And YI, our indirectly associated phenotypes, have a connection with g only by going through YD first. Note that if YD were not observed, YI would appear as a direct connection. (PDF) [file pgen.1008431.s001.pdf]

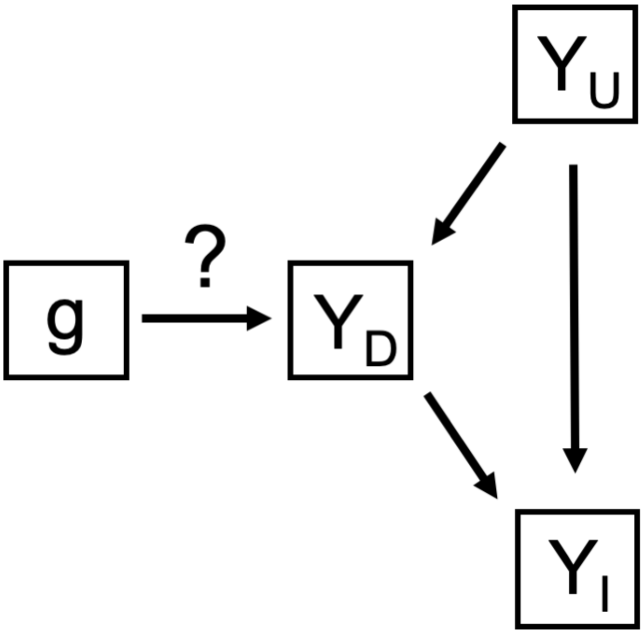

S1 Fig. Graphical Model of Multivariate Categories.
Shown here is a Directed Acyclic Graphical (DAG) model of our multivariate categories in the context of our vector of phenotypes $\mathbf{Y}$ (e.g. $\mathbf{Y} = \{\mathbf{Y_U}, \mathbf{Y_D}, \mathbf{Y_I}\}$) and their connections with the variant of interest $\mathbf{g}$. The relationships described in-text can be seen here. $\mathbf{Y_U}$, our unassociated phenotypes, have no connection with $\mathbf{g}$. $\mathbf{Y_D}$, our directly associated phenotypes, have a direct connection with $\mathbf{g}$. And $\mathbf{Y_I}$, our indirectly associated phenotypes, have a connection with $\mathbf{g}$ only by going through $\mathbf{Y_D}$ first. Note that if $\mathbf{Y_D}$ were not observed, $\mathbf{Y_I}$ would appear as a direct connection.
