# Supplementary material for: Bayesian multivariate reanalysis of large genetic studies identifies many new associations
Source: PLoS Genet. 2019 Oct 9;15(10):e1008431. doi: 10.1371/journal.pgen.1008431 (PMC6802844; doi:10.1371/journal.pgen.1008431)
Supplement: S2 Fig — Shown are the -log10 univariate p-values from the GlobalLipids2013 analysis for both the previous univariate association rs7515577 (“Previous Univariate SNP”) and the new multivariate association rs12038699 (“New Multivariate SNP”) across all four phenotypes analyzed. rs7515577 is represented as a triangle and rs12038699 is represented as a square. Also shown are the -log10 univariate p-values of SNPs within 1Mb of the midpoint between rs7515577 and rs12038699. Color-coding of the SNPs represent the degree of linkage disequilibrium between variants and the new association rs12038699 based on the GBR cohort of 1000Genomes [49]; for color coding details, see legend. (PDF) [file pgen.1008431.s002.pdf]

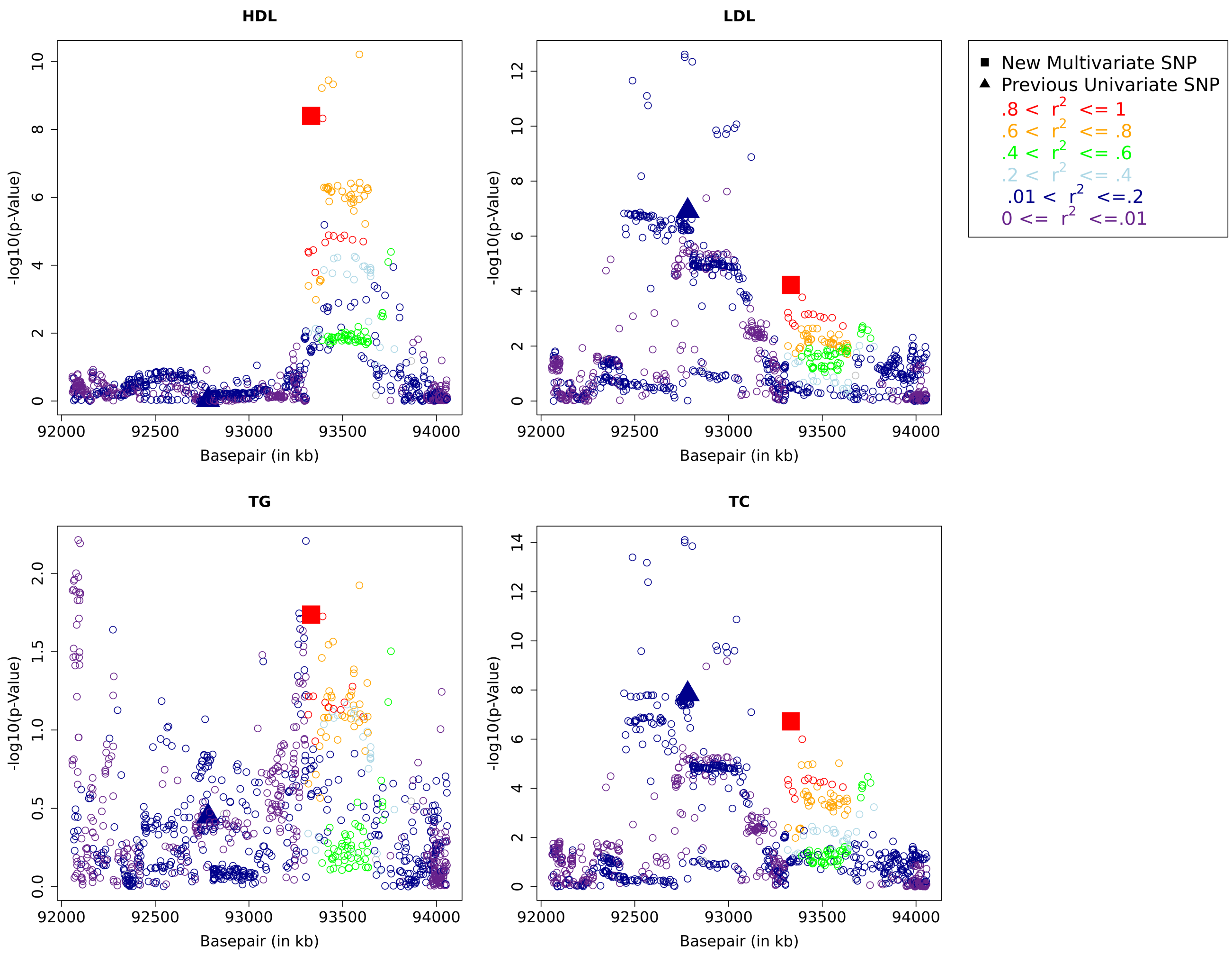

S2 Fig. Refining Association Signals – GlobalLipids2013 rs7515577 & rs12038699.
Shown are the -$\log_{10}$ univariate p-values from the GlobalLipids2013 analysis for both the previous univariate association rs7515577 ("Previous Univariate SNP") and the new multivariate association rs12038699 ("New Multivariate SNP") across all four phenotypes analyzed. rs7515577 is represented as a triangle and rs12038699 is represented as a square. Also shown are the -$\log_{10}$ univariate p-values of SNPs within 1Mb of the midpoint between rs7515577 and rs12038699. Color-coding of the SNPs represent the degree of linkage disequilibrium between variants and the new association rs12038699 based on the GBR cohort of 1000Genomes (Genomes Project et al., 2015); for color coding details, see legend.
