# Supplementary material for: Bayesian multivariate reanalysis of large genetic studies identifies many new associations
Source: PLoS Genet. 2019 Oct 9;15(10):e1008431. doi: 10.1371/journal.pgen.1008431 (PMC6802844; doi:10.1371/journal.pgen.1008431)
Supplement: S3 Fig — Shown are the phenotype effect sizes (points), and ±2 standard errors (bars), for four significantly associated SNPs from HaemgenRBC2016. All four SNPs were classified as being “associated” with all six phenotypes (i.e. marginal posterior probability of association >= 95% for each phenotype). However, they clearly show different patterns of effect sizes. Therefore focusing simply on binary calls of “associated” vs “unassociated” can hide different patterns of multivariate association. (PDF) [file pgen.1008431.s003.pdf]

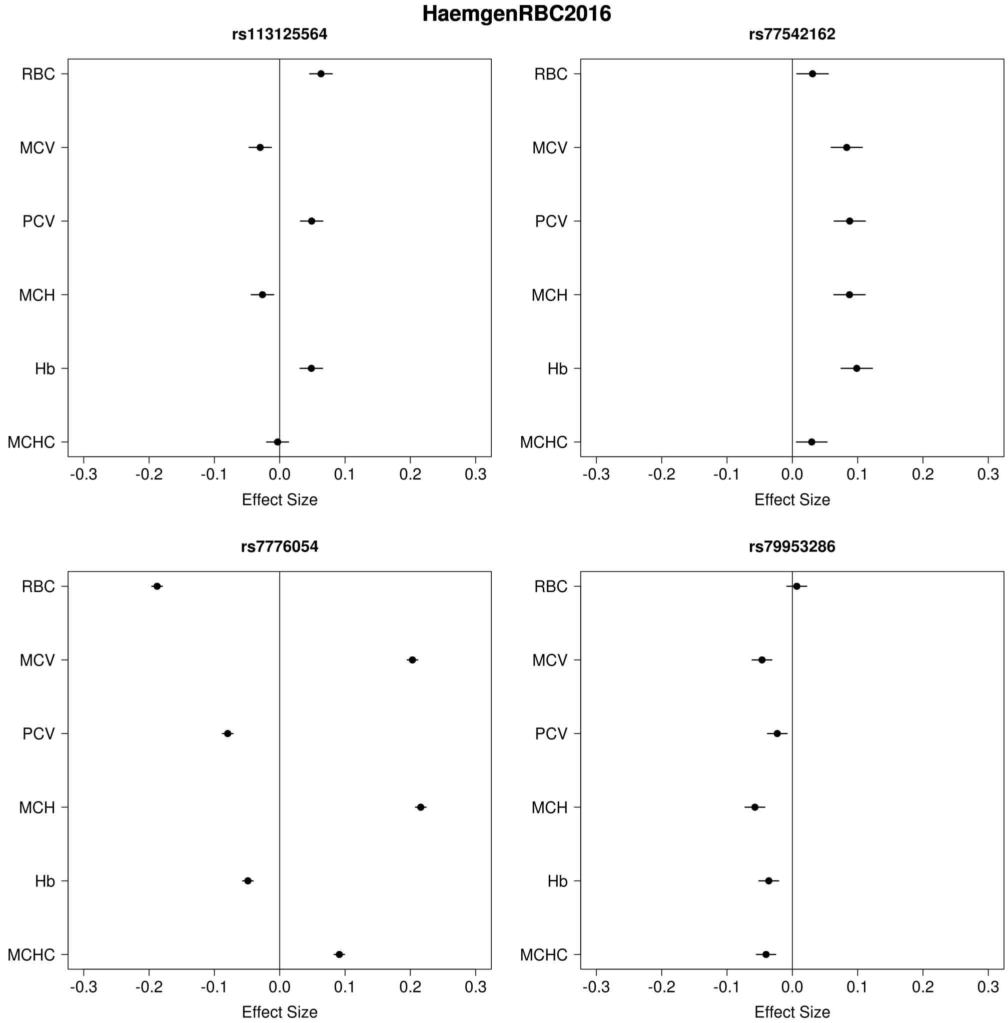

S3 Fig. Effect Size Heterogeneity Among SNPs With Identical Multivariate Model Assignments.
Shown are the phenotype effect sizes (points), and ±2 standard errors (bars), for four significantly associated SNPs from HaemgenRBC2016. All four SNPs were classified as being "associated" with all six phenotypes (i.e. marginal posterior probability of association >= 95% for each phenotype). However, they clearly show different patterns of effect sizes. Therefore focusing simply on binary calls of "associated" vs "unassociated" can hide different patterns of multivariate association.
