# Supplementary material for: Bayesian multivariate reanalysis of large genetic studies identifies many new associations
Source: PLoS Genet. 2019 Oct 9;15(10):e1008431. doi: 10.1371/journal.pgen.1008431 (PMC6802844; doi:10.1371/journal.pgen.1008431)
Supplement: S1 Table — a Number of new multivariate associations discovered by our analysis. Note that we required a multivariate association to be at least 500kb from a previous reported association to be considered “new”. b Univariate GWAS significance p-value threshold used by the original study publication. c These are new multivariate SNPs that were not reported by the original study despite having a univariate association (in the public summary data) that was genome-wide significant by the original study’s univariate significance threshold. d A “previous association” means an association reported by the original GWAS; “near” means within 1Mb (but these are all more than 500kb away from a previous association since our classification of new multivariate SNPs requires this). (PDF) [file pgen.1008431.s004.pdf]

S1 Table. Summary of Associations in Each Dataset.

Dataset	Release	New Multivariate SNPs[a]	Univariate p-Value Threshold[b]	New SNPs Below Univariate Threshold[c]	New SNPs Near Previous Association[d]
GlobalLipids	2010	19	5×10^{-8}	0	0
	2013	65	5×10^{-8}	18	11
GIANT	2010	60	5×10^{-8}	6	5
	2014/5	162	5×10^{-8}	41	21
HaemgenRBC	2012	16	1×10^{-8}	4	3
	2016	60	8.31×10^{-9}	0	0
ICBP	2011	22	5×10^{-8}	2	1
MAGIC	2010	1	5×10^{-8}	0	0
GEFOS	2015	13	1.2×10^{-8}	3	1
GIS	2014	5	5×10^{-8}	5	0
SSGAC	2016	1	5×10^{-8}	0	0
CKDGen	2010/1	6	5×10^{-8}	0	0
ENIGMA2	2015	3	7.1×10^{-9}	0	0

[a] Number of new multivariate associations discovered by our analysis. Note that we required a multivariate association to be at least 500kb from a previous reported association to be considered "new".

[b] Univariate GWAS significance p-value threshold used by the original study publication.

[c] These are new multivariate SNPs that were not reported by the original study despite having a univariate association (in the public summary data) that was genome-wide significant by the original study's univariate significance threshold.

[d] A "previous association" means an association reported by the original GWAS; "near" means within 1Mb (but these are all more than 500kb away from a previous association since our classification of new multivariate SNPs requires this).
