# Supplementary material for: Bayesian multivariate reanalysis of large genetic studies identifies many new associations
Source: PLoS Genet. 2019 Oct 9;15(10):e1008431. doi: 10.1371/journal.pgen.1008431 (PMC6802844; doi:10.1371/journal.pgen.1008431)
Supplement: S5 Table — Shown are example metrics of how well our new multivariate associations replicate in datasets that allow such an evaluation. Specifically, for three of the studies used (GlobalLipids, GIANT, and HaemgenRBC), there are multiple dataset releases. To examine how well our new multivariate bmass associations replicate, we compared the results from the first releases (“1st”) with the univariate GWAS associations of the second releases (“2nd”). In essence, each of these approaches aim to increase power—one by using a multivariate approach (bmass) and the other by increasing sample size (the 2nd releases)—thus allowing us to compare the results against one another. Univariate p-Value Threshold: univariate GWAS significance p-value thresholds used by the original publication(s) for both the earlier (1st) and later (2nd) releases. New Multivariate SNPs in 1st: number of new multivariate associations from the earlier release. Lower Univariate p-Value in 2nd: number of new multivariate associations from the earlier release that also have lower univariate p-values in the later release. Below 2nd Univariate Threshold: number of new multivariate associations from the earlier release that also cross the later release’s univariate GWAS significance threshold. (PDF) [file pgen.1008431.s008.pdf]

**Dataset**	**Releases (1st, 2nd)**	**Univariate *p*-Value Threshold (1st, 2nd)**	**New Multivariate SNPs in 1st**	**Lower Univariate *p*-Value in 2nd**	**Below 2nd Univariate Threshold**
GlobalLipids	2010, 2013	5×10^{-8} ,5×10^{-8}	19	14	11
GIANT	2010, 2014/5	5×10^{-8}, 5×10^{-8}	60	59	51
HaemgenRBC	2012, 2016	1×10^{-8}, 8.31×10^{-9}	16	11	6

S5 Table. Replication of New Multivariate Associations.
Shown are example metrics of how well our new multivariate associations replicate in datasets that allow such an evaluation. Specifically, for three of the studies used (GlobalLipids, GIANT, and HaemgenRBC), there are multiple dataset releases. To examine how well our new multivariate `bmass` associations replicate, we compared the results from the first releases ("1st") with the univariate GWAS associations of the second releases ("2nd"). In essence, each of these approaches aim to increase power – one by using a multivariate approach (`bmass`) and the other by increasing sample size (the 2nd releases) – thus allowing us to compare the results against one another. Univariate *p*-Value Threshold: univariate GWAS significance *p*-value thresholds used by the original publication(s) for both the earlier (1st) and later (2nd) releases. New Multivariate SNPs in 1st: number of new multivariate associations from the earlier release. Lower Univariate *p*-Value in 2nd: number of new multivariate associations from the earlier release that also have lower univariate *p*-values in the later release. Below 2nd Univariate Threshold: number of new multivariate associations from the earlier release that also cross the later release's univariate GWAS significance threshold.
