# Supplementary material for: Bayesian multivariate reanalysis of large genetic studies identifies many new associations
Source: PLoS Genet. 2019 Oct 9;15(10):e1008431. doi: 10.1371/journal.pgen.1008431 (PMC6802844; doi:10.1371/journal.pgen.1008431)
Supplement: S6 Table — In the 2010 release rs7515577 has a univariate p-value that crosses the 5 × 10^−8^ threshold (TC), whereas rs12038699 does not. Since rs12038699 is near to rs7515577 it may get masked for future analyses; however in the 2013 data rs12038699 not only has a lower minimum univariate p-value, but also has a different multivariate p-value pattern as compared to rs7515577. Both these signals suggest that rs12038699 should be viewed as a separate GWAS hit for GlobalLipids2013. (PDF) [file pgen.1008431.s009.pdf]

SNP	Phenotype	Direction[a]	2010[b]	2013[c]
**rs7515577**				
(Previous)	HDL	+	9.81E-01	9.29E-01
	LDL	-	1.51E-07	1.21E-07
	TG	-	1.80E-01	3.57E-01
	TC	-	2.78E-08	1.47E-08
**rs12038699**				
(New)	HDL	+	4.22E-05	3.98E-09
	LDL	+	1.06E-03	5.95E-05
	TG	+	8.51E-02	1.83E-02
	TC	-	7.12E-05	1.90E-07

[a] Whether the reference allele increases (+) or decreases (-) phenotype.
[b] *p*-value from GlobalLipids 2010.
[c] *p*-value from GlobalLipids 2013.

S6 Table. *p*-Values for rs7515577 & rs12038699 in 2010 and 2013 GlobalLipds Releases.
In the 2010 release rs7515577 has a univariate *p*-value that crosses the 5×10^{-8} threshold (TC), whereas rs12038699 does not. Since rs12038699 is near to rs7515577 it may get masked for future analyses; however in the 2013 data rs12038699 not only has a lower minimum univariate *p*-value, but also has a different multivariate *p*-value pattern as compared to rs7515577. Both these signals suggest that rs12038699 should be viewed as a separate GWAS hit for GlobalLipids2013.
