# Supplementary material for: Bayesian multivariate reanalysis of large genetic studies identifies many new associations
Source: PLoS Genet. 2019 Oct 9;15(10):e1008431. doi: 10.1371/journal.pgen.1008431 (PMC6802844; doi:10.1371/journal.pgen.1008431)
Supplement: S7 Table — List of multivariate models that most frequently have the highest posterior probabilities per SNP. Top 5 models are shown from across both the previous univariate associations analyzed and the new multivariate associations discovered in the GlobalLipids2013, GIANT2014/5, and HaemgenRBC2016 datasets. Phenotype ordering is shown in the header, where 0, 1, and 2 refer to the multivariate categories of Unassociated, Directly Associated, and Indirectly Associated. n is the number of SNPs that show the specified model as having the largest posterior probability, with Mean Posterior displaying the average posterior probability of the given model across the n SNPs, and Original Prior showing the prior established for the given model from training on all the previous univariate associations from that dataset. (PDF) [file pgen.1008431.s010.pdf]

**GlobalLipids2013: New+Prev SNPs**				
	**HDL_LDL TG_TC**	**n**	**Mean Posterior**	**Original Prior**
	1_2_1_1	100	0.635	0.327
	1_2_2_1	38	0.531	0.138
	1_1_1_2	18	0.751	0.117
	2_1_2_2	17	0.661	0.057
	1_1_1_1	11	0.769	0.078
**GIANT2014/5: New+Prev SNPs**				
	**Height_BMI WHRadjBMI**	**n**	**Mean Posterior**	**Original Prior**
	1_2_0	616	0.443	0.318
	1_1_1	143	0.677	0.161
	1_1_0	89	0.488	0.094
	1_2_1	15	0.387	0.037
	1_2_2	13	0.623	0.257
**HaemgenRBC2016: New+Prev SNPs**				
	**RBC_MCV_PCV MCH_Hb_MCHC**	**n**	**Mean Posterior**	**Original Prior**
	2_1_1_2_2_2	179	0.487	0.17
	2_1_2_2_1_1	162	0.502	0.203
	2_1_0_2_2_1	105	0.498	0.117
	2_1_1_2_2_1	51	0.561	0.155
	2_0_1_2_2_2	33	0.405	0.038

S7 Table. Top Multivariate Model Examples per SNP.
List of multivariate models that most frequently have the highest posterior probabilities per SNP. Top 5 models are shown from across both the previous univariate associations analyzed and the new multivariate associations discovered in the GlobalLipids2013, GIANT2014/5, and HaemgenRBC2016 datasets. Phenotype ordering is shown in the header, where 0, 1, and 2 refer to the multivariate categories of **U**nassociated, **D**irectly Associated, and **I**ndirectly Associated. n is the number of SNPs that show the specified model as having the largest posterior probability, with Mean Posterior displaying the average posterior probability of the given model across the n SNPs, and Original Prior showing the prior established for the given model from training on all the previous univariate associations from that dataset.
