# Supplementary material for: Bayesian multivariate reanalysis of large genetic studies identifies many new associations
Source: PLoS Genet. 2019 Oct 9;15(10):e1008431. doi: 10.1371/journal.pgen.1008431 (PMC6802844; doi:10.1371/journal.pgen.1008431)
Supplement: Attachment — Submitted filename: TurchinStephens2019_ReviewerResponse2.pdf [file pgen.1008431.s011.pdf]

Response to reviewers: Bayesian multivariate reanalysis of large genetic studies identifies many new associations

Michael C. Turchin and Matthew Stephens

July 2019

Comments by Reviewer #1

> Turchin & Stephens show that many more associations can be identified with their Bayesian multivariate analysis. This paper advocates an important message that statistical power can easily improved from univariate to multivariate analysis.
>
> Overall the paper is well written and results are striking. One interesting question is that whether the performance gain is from the bayesian framework (i.e., bmass) or from multivariate analysis, a simple way to address that is to apply another multivariate method and compare its results with that from bmass.

Thank you for your very positive comments.

We feel that one of the strengths of our manuscript is that it has a very simple message: that multivariate analysis can be substantially more powerful than univariate analysis, and that multivariate analysis is underused in practice. We would prefer not to risk diluting this message by getting distracted by a different issue: the relevant advantages and disadvantages of different multivariate methods. Indeed, we feel that it would be impossible to do this issue justice by minor modifications to this paper, and note that whole papers have already been written devoted to comparing different multivariate analysis approaches (e.g. Galesloot et al. 2014; Porter and O'Reilly 2017). Also, the original publication describing the method we use here devotes a lot of space to addressing the relative benefits of the

Bayesian approach compared with simple multivariate methods (Stephens, 2013). Therefore, we decided not to add additional material on this topic here.

Comments by Reviewer #2

> Overview: This manuscript takes the much needed step to show consortiums what they miss by not considering multivariate analysis of related phenotypes. The authors re-analyzed several publicly available datasets to compare findings from multivariate analysis of multiple phenotypes against those from single-trait analyses. Further, the authors empirically validate their additional findings from multivariate analysis by replicating those findings in larger, more recent datasets (e.g. in studies with multiple releases where later releases have larger sample sizes). The authors additionally emphasize that multivariate analysis is different from multiple single-trait analyses.
>
> I have a few discussion-type comments that will likely help other researchers adopt multivariate analyses in GWAS (which seems to be the goal of this manuscript) and a few minor comments for the authors to consider.

Thank you for your very positive comments.

> *Major comments*
>
> 1. The authors give a couple reasons for why multivariate analyses are not adopted more widely. One reason that I have encountered from consortiums is that two traits may have widely different sample sizes, and no one wants to "sacrifice" higher sample size of one trait by trying to jointly analyze the two traits. This idea is probably stemming from the fact that multivariate analysis based on individual-level data requires the two traits to be measured on the same set of individuals. So, I think it will be useful to emphasize that these days, multivariate analysis can be done using methods based on GWAS summary statistics that do not have such a requirement. However, it also comes with additional questions that needs discussion:
> (a) if the consortiums have individual-level phenotype-genotype data where each trait has widely varying sample sizes, should the analysts first obtain single-trait single-SNP GWAS summary statistics and then proceed with multivariate analysis (using methods based on summary data)? Is this 2-step process an efficient approach? What would the authors recommend?
> (b) are the multivariate methods based on summary data robust across wide differences in sample sizes? Or can the authors give a thumb-rule (that analysts can follow) on the ratio of sample sizes of the traits beyond which summary-data-based-multivariate-methods may not be robust and may not prove more effective than univariate analysis?

We appreciate the reviewer's additional insights into reasons consortia do not readily adopt multivariate approaches. We do want to clarify that although our methods work with summary data, the original formal derivation of the methods assumes the data come from the same individuals. So although the software could be run on data from different individuals as the referee suggests, one has to be a bit careful (particularly in interpreting direct vs indirect effects). Consequently we limited our analysis here to datasets on traits measured on the same individuals (or at least heavily overlapping sets of individuals).

That said, because the method uses an empirical approach to assessing the correlations among the null Z-scores, we do expect it to be somewhat robust to this issue. We have now included an explicit discussion of this in the manuscript.

a) Yes, the setup the reviewer outlines is indeed our recommendation. The main reason being that this is a very flexible approach: researchers will have a greater ability to conduct a wide range of analyses by first doing univariate analysis on the individual-level data (if available). In particular our current framework does not immediately have options for including covariates in the multivariate analysis, so correcting for confounders such as population structure should be done on the first

set of univariate analyses. We have added specific mention of this to the Discussion.

b) We speculate the answer to the reviewer's first question in (b) is generally 'yes', but this is a potentially subtle issue (e.g. some multivariate methods may be more robust than others) and we do not feel comfortable giving a 'thumb-rule' without a complete investigation, which lies outside the scope of this paper.

> In my opinion, another hurdle in the adoption of multivariate analysis in consortiums: usually the multivariate analyses focus on genetic variants with MAF 1% or more (due to concerns about type I error inflation) while univariate analyses can conveniently go to lower MAFs without as much worry about inflation. Consequently, univariate analyses may end up detecting significantly associated genetic regions that are not even considered for multivariate analyses in the first place (due to more stringent MAF screening). Apparently then multivariate analysis is not giving anything over and above the findings from univariate analysis - the end result is the publication of univariate analysis results only. How would the authors sell multivariate methods to consortiums in such a context where univariate analysis appears more effective?

We agree that multivariate analysis can be sensitive to very low minor allele frequency, so we would agree that it is prudent to impose an MAF cut-off on the multivariate analyses. We do not see any problem with doing this in addition to the univariate analyses (with a less stringent MAF cut-off), and we would expect the multivariate analysis to generally produce many additional hits (as it does in our analyses here). Under the scenario where a consortium finds that running a multivariate analysis does not indicate any new SNPs as being genome-wide significant, then we would not criticize them for choosing to focus on univariate analyses. We have added a discussion of this to the manuscript.

> 2. Since the authors are advocating multivariate analyses (which I completely agree with), I think readers will find it useful if the authors can provide a general multivariate analysis pipeline that consortiums can broadly follow. For instance, a general univariate analysis pipeline involves (1) linear or logistic regression of each trait, (2) then clumping of significant variants into unique loci, (3) then performing conditional analyses to determine which signals are independent from signals that are known from past studies, (4) then trying to find biological relevance of independent new signals for the corresponding trait, etc. On the other hand, for multivariate analysis, to avoid the problem of sacrificing higher sample size of one trait (as pointed out in the previous comment), (1) the first step will still be univariate analysis, (2) then multivariate analysis using say BFav approach based on the summary stats from first step (is BFav only applicable to continuous traits?), (3) then clumping of unique loci, (4) then performing conditional analyses? How to do conditional analyses with BFav approach? Which SNP-trait associations from past studies should be considered for conditional analyses? For the 5th step, how should one go about determining biological relevance of a multivariate signal? What next steps can be taken?

We agree with the reviewer that having well-detailed and reproducible pipelines in general is a benefit to the community and likely to help with multivariate method adoption. In our software package `bmass` (https://github.com/mturchin20/bmass) we provide two vignettes that give examples of how researchers may conduct such multivariate analyses. One vignette illustrates `bmass` using simulated data, and the other is a more advanced tutorial starting with the GlobalLipids2013 data – in this second vignette, we do indeed show users a basic pipeline on how to go from downloaded data to multivariate analysis in R (including some basic QC and data harmonization). This example pipeline provides a starting point for readers wanting to use the method themselves. We have included an explicit mention to these vignettes in the Methods section.

Regarding conditional analysis, we view this approach as a first step towards multivariate fine-mapping of association signals, and this is an area that we believe requires more work (indeed, there are very few multivariate methods for fine mapping). We have added a mention of this in the Discussion.

Regarding determining biological relevance, we feel how to best understand the biological relevance of even univariate GWAS is, of course, a complex topic. We do not see the multivariate case to be much different than the univariate case here, although arguably there are some advantages of multivariate approaches which we already addressed in the Discussion (paragraph beginning "Moving forward, we expect multivariate association analyses to play an increasingly important

role in detecting and understanding genetic associations and relationships among phenotypes."). We therefore have not added anything further.

> 3. The authors defined "new multivariate association" as significant "if its BFav exceeds that of any previous univariate association's BFav in the same study (Stephens, 2013). The rationale is that the evidence for these multivariate associations exceeds the evidence for univariate associations that are generally accepted as likely to be real." - Is this, in some sense, equivalent to (in the frequentist world) defining significance by looking at the multivariate p-value; if it is smaller than the minimum of the univariate p-values, then we have a new multivariate association?
> If so, are we going to call this situation new multivariate association: p_Trait1 = 0.01, p_Trait2=0.005 and p_multivariate=0.001?
> If yes, the definition does not seem right because in GWAS, we look at more stringent significance thresholds. Can the authors explain in more detail?

We think the reviewer misunderstood: 'previous univariate association' refers to **genome-wide significant** univariate associations. So we are comparing the multivariate evidence at each SNP with the multivariate evidence at previously-reported genome-wide significant SNPs (not with the univariate evidence at the same SNP). So the situation described by the reviewer would not be considered a new multivariate association. We have edited the manuscript to emphasize that the comparison is with previous genome-wide significant associations.

> 4. Figure 2: Some of the new multivariate SNPs are below the 45-degree line. If I am interpreting it correctly, these SNPs have larger negative log-transformed p-values (i.e., more significant) in the earlier release than the later release (later release with larger sample size). When can this happen? Are these variants low-frequency?

Yes, the reviewer is interpreting the plot correctly – these SNPs have more significant p-values in the earlier releases than compared to the later releases. This could suggest that the original association was not real (false positive), but it is also not unexpected for real associations in situations where power is incomplete (as in GWAS). Additionally, it does not have to be due to low MAF. The intuition is that even real associations can have "non-significant" results in a replication sample (not significant does not imply not real) and this could cause the p-value in the combined sample to be lower than in the original study.

> *Minor Comments*
> 1. Since the authors recommend re-analyzing published GWAS using multivariate methods that use single-SNP summary data, I think it is worth emphasizing that consortiums ensure detailed summary data files are made publicly available. For instance, I have often come across publicly available summary data files that are almost useless because they have missing MAF or risk allele frequency, or missing info on the risk allele or the reference allele, or missing info on the Z statistics (or beta estimates) making direction of effect indeterminable.

We whole-heartedly agree and commiserate with the reviewer's comment! We have added this to the Discussion section.

> 2. "in total, 84 of 94 new associations have smaller univariate p-values in the later release, and indeed the majority of these reach univariate GWAS significance in the later release." - Do the authors mean exactly the same variants have smaller univariate p-values in later release compared to the first release?

Yes. We have edited the sentence to help clarify this.

> 3. "at a univariate threshold of 5e-7 only 66% of the univariate significant SNPs are also multivariate significant across these three studies." - Was 5e-7 threshold used for determining multivariate significance as well? What's the equivalent BFav value (since the multivariate approach used the BFav Bayesian approach)?

Here multivariate significant means a BF_{av} exceeding our significance threshold, which as previously stated is based on the BF_{av} values of previous univariate GWAS significant SNPs. We have clarified this by saying "multivariate significant *in our analysis*" to make it clear that we are not introducing a new notion of multivariate significant here, but using the same notion as earlier in the paper.

> 4. Can the authors explain the following choices?
>
> (a) Looks like the author screened our variants with MAF $<$ 1% for all datasets but one. The MAF threshold for GEFOS2015 is 0.5%.
>
> (b) "We used a minimum N threshold of 50,000".
>
> (c) The authors used "a univariate significant GWAS p-value threshold of 5e-8" for most studies but not all (e.g., for HaemgenRBC2016, threshold used is 8.319e-9).

a) In general we tried to match QC steps to the original publication. In most cases these studies used MAF thresholds of 1%, but GEFOS2015 used 0.5%. Some studies used very rare MAF thresholds, and for those we applied the 1% threshold as multivariate results for very low frequency variants may not be reliable.

b) This was just a simple basic QC step to remove SNPs that had particularly high missingness rates.

c) We used the univariate GWAS p-value threshold as detailed by the original publications; not every publication used 5×10^{-8}.

> 5. "There may be many reasons why such variants went unreported, but one reason may be physical proximity to a variant with a stronger signal." - Is it possible that such variants were not reported because they were no longer significant in subsequent conditional analyses?

Indeed this could be one of the 'many reasons' alluded to in the above sentence. However, it seems that such an extra step is generally considered 'follow-up' and is explicitly detailed, versus say LD-based pruning which is viewed more as a fundamental part of the basic GWAS pipeline. And not all studies make mention of conducting such conditional analyses in their associated publications. So our guess is no, but we do not know.

> 6. Supplementary Figure 3 plots effect sizes for each trait. Does the BFav approach provide effect size estimates as well?

Not as currently implemented – our main focus with this framework was assigning multivariate associations to the best-supported, underlying **U**,**D**,**I** models. One could get Bayesian estimates of effect sizes, e.g. as in Urbut et al. 2019, but we have not yet implemented this in `bmass`.

Comments by Reviewer #3

> Reviewer #3: This is a nicely written paper applying a multivariate method to 13 datasets to search for new associations. The paper presents a large number of new findings, with the ultimate goal of encouraging increased use of the multivariate method.

Thank you for your very positive comments.

> General comments:
>
> The performance of this multivariate approach is illustrated by looking at very closely related phenotypes, such as blood cell subtypes and anthropometric measures. Would this method still yield substantial power gains when investigating joint associations for less similar traits (i.e.: BMI and different cancer types)? Is the power of this approach related to the degree of correlation (genetic and/or phenotypic) between the traits?

This issue is quite subtle, and discussed extensively in Stephens 2013. In brief, multivariate analysis is most helpful for correlated traits, whether or not they share genetic factors in common. Indeed, somewhat surprisingly it is most helpful when they do not share genetic factors in common. For (almost) uncorrelated traits, multivariate analysis is still helpful if genetic factors are shared, but not very helpful if genetic factors are independent.

> The authors note that "we declared a multivariate association as significant if its BFav exceeds that of any previous univariate association's BFav in the same study." Thus, the multivariate is deemed significant if it has a Bayes factor larger than any one univariate result. Is this criterion too liberal, and might it result in false positives for the multivariate approach?

To be clear, a multivariate SNP is deemed significant if it has a Bayes Factor larger than any one **genome-wide significant** univariate result – meaning we deem any new SNP significant only in comparison to the univariate SNPs that were already accepted as significant. In other words, you can think of this as our attempt to transform the conventional univariate significant p-value threshold (e.g. 5×10^{-8}) into our 'Bayesian multivariate space'; the least significant univariate GWAS SNP will have the p-value closest to this univariate threshold, so therefore this least significant SNP's BF_{av} will be the value that's closest as possible to the BF_{av} that's equivalent to the univariate threshold. How you feel about whether

this choice of BF_{av} threshold is too liberal or conservative then should mostly be related to how you feel about the original univariate threshold used. However, we feel it is a reasonable pragmatic approach, and our analysis of replication in studies with multiple releases suggest that it is not resulting in large numbers of false positives.

> It is unclear why examining associations "that would be declared significant if the univariate significance threshold were relaxed" and comparing these to multivariate association results illustrates that multivariate analysis is not the same as multiple univariate analyses. I agree that these are not the same thing, but wouldn't it make sense to look at whether a given SNP is associated with traits 1-3 in multiple univariate analyses and then see if it is also identified in the multivariate analysis of the same traits?

We believe that what the reviewer suggests is indeed what we are doing in this analysis. For this particular analysis, we gradually lower the univariate significance threshold and collect the SNPs which now have univariate associations (for at least one trait) below this more relaxed threshold. We then ask whether, amongst these new associations (i.e. that were found by looking at multiple univariate analyses), they overlap with the list of new multivariate associations we have already identified. Therefore we are indeed directly comparing SNPs that were discovered by conducting multiple univariate analyses to SNPs that were discovered by conducting multivariate analysis.

To try to clarify this we have added an explanatory comment to our text describing the method: "(i.e. we assess whether, if we go deeper into the univariate results, we find the same SNPs as appear in our multivariate results)."

> The "Reanalysis also identifies new univariate associations" part is a little vague. Does this mean that there were significant SNPs in the original summary stats files that were simply not reported? Or are these new SNPs that were identified in the multivariate analysis, but were only associated with one trait?

Yes, the former explanation is correct: these are significant SNPs in the original summary statistics files that were not reported. We agree this is somewhat mysterious, but it is true nonetheless. We have edited the first paragraph of this section to make this clearer.

> Showing replication is very important to support the use of the multivariate approach. When using a later release of the same study/consortium to replicate associations, were the older studies excluded from the new release? What is the degree of overlap between the two, and can this be considered independent replication? If the initial study is included in the latter release, wouldn't one expect the univariate p-value to decrease based on the larger sample size regardless of it being a true signal?

To answer the first question, no, the older studies were not excluded: later releases generally include all (or almost all) of the previous release samples. That is why we focus on whether the p-value is more significant in the later release than the original release (Figure 2), because this measures whether the signal in the new (non-overlapping) samples provides additional evidence *over and above* the original signal.

Regarding the second question, the answer is also no: a larger sample size does not lead to smaller p-values if there is no signal. It might help the reviewer to think about averaging Z-scores from two samples: if the first study shows a big Z-score, but the second study shows a non-significant Z-score, then averaging it with the Z-score from the first study can make things less significant. Another way of thinking: while under the alternative hypothesis of association a larger sample size generally leads to greater power, under the **null hypothesis** there is no effect of sample size (p-values are always uniform under the null, independent of sample size). In fact even if there is a signal the p-value could get less significant in the larger sample (both due to chance and due to winner's curse issues).

A full discussion of these issues lies outside the scope of the paper, but we have added text to briefly clarify these points.

> When evaluating the independence of new multivariate associations, it seems that pruning was based on distance (SNPs at least 0.5 Mb apart), but was LD was explicitly considered based on an r2 cut-off?

No, since we were using summary data and not direct genotypes we were unable to prune via LD. However we feel that our approach is likely to be more conservative than using LD, so we are at least controlling for false positives with this assumption.

> Why were different significance thresholds used for different studies? HaemgenRBC 2012: p<110-8, HaemgenRBC 2016: p<8.31910-9, GEFOS2015: p<1.210-8

We used the original genome-wide univariate significant thresholds used by each study; so these are the p-value thresholds used in each of the respective publications. They did not all use the same threshold since choice of suitable GWAS threshold remains somewhat subjective.

> In the Reanalysis section the authors state "Indeed the very different multivariate patterns of effect size suggest that not only are these associations non-redundant but likely involve different biological mechanisms as well." Is there evidence to support this statement? Do we really think that different effect sizes mean different mechanisms?

We think we were unclear: indeed, we did not intend to claim that different **effect sizes** imply different mechanisms. Rather we meant different **patterns of effect sizes** and particularly different directions/signs of effects. For example, imagine an allele at SNP 1 increases HDL and lowers LDL, whereas an allele at SNP 2 increases both. It is hard to imagine that these two SNPs are operating through the same biological mechanism. We have modified the text to clarify this point.

Response to Editor

> I noted that you mention "unpublished" on page 27, line 409. Please note that PLOS Genetics recently updated its data access policy and now requires that all data be either published with the manuscript or made available in a publically accessible database. We ask that you either amend the supplementary material to include the referenced data or remove as many of the references as possible. Please include an explanation of how you have met this request (i.e. whether you have simply removed the instances because they are not integral to the manuscript, or whether you have provided further supporting information files) at the end of your response to reviewers. Please note that we will not be able to pass your manuscript through our technical checks unless this is accompanied by this explanation.

Our use of "unpublished" on page 27, line 409 is in reference to the previous studies we collected data from. We are classifying some of our current results as possibly having been 'unpublished' from the previous studies (eg results that possibly should have been published by the previous studies but for some unknown reasons were not) – so we are not actually discussing results that we, ourselves, are

keeping unpublished. Our results are still shared. We hope this clarifies our use of "unpublished".

References

Galesloot, T. E., van Steen, K., Kiemeney, L. A., Janss, L. L., and Vermeulen, S. H. (2014). A comparison of multivariate genome-wide association methods. *PLoS One*, 9(4):e95923.

Porter, H. F. and O'Reilly, P. F. (2017). Multivariate simulation framework reveals performance of multi-trait gwas methods. *Sci Rep*, 7:38837.

Stephens, M. (2013). A unified framework for association analysis with multiple related phenotypes. *PLoS One*, 8(7):e65245.

Urbut, S. M., Wang, G., Carbonetto, P., and Stephens, M. (2019). Flexible statistical methods for estimating and testing effects in genomic studies with multiple conditions. *Nat Genet*, 51(1):187–195.
