# Supplementary material for: Bayesian multivariate reanalysis of large genetic studies identifies many new associations
Source: PLoS Genet. 2019 Oct 9;15(10):e1008431. doi: 10.1371/journal.pgen.1008431 (PMC6802844; doi:10.1371/journal.pgen.1008431)
Supplement: Attachment — Submitted filename: TurchinStephens2019_ReviewerResponse_Rnd2_v2.pdf [file pgen.1008431.s012.pdf]

Response to reviewers v2: Bayesian multivariate reanalysis of large genetic studies identifies many new associations

Michael C. Turchin and Matthew Stephens

September 2019

Comments by Reviewer #2

> Reviewer #2: I thank the authors for addressing my previous comments. With regards to the authors' "response to reviewers", it would have been less time intensive to review if the authors highlighted the text they added in the manuscript. Or, at least mention the relevant page numbers wherever "We have now included [...] in the manuscript" is mentioned in their response. I'd request the authors to consider giving this info and/or an annotated manuscript in any subsequent review.

We apologize for the additional effort the lack of such annotations caused – we agree with the reviewer and will make more of an effort to include specific references to edits moving forward.

> *Minor Comments* 1. The authors added "More generally, although not necessarily essential for our analyses here, it is always helpful to include additional key meta-data (e.g. the reference allele, or effect allele, the minor allele frequency, and sample size)." However, earlier the authors mentioned in section 6.1 that they dropped variants from GWAS datasets that had missing MAF or for which reference/alt alleles did not match across phenotypes. So, I am not sure why the authors say meta-data such as "reference allele, or effect allele, the minor allele frequency" are "not necessarily essential" for their analyses. It may not be necessary for implementing their R program bmass but these info seem essential for subsequent interpretation (without which, there's high risk of erroneous conclusions about statistical significance - e.g., mixing up effect alleles across traits can inflate results; results may not be trustworthy if the MAF is low/rare).

We agree with the reviewer that the wording here is not clear. We have removed "although not necessarily essential for our analyses here" from lines 277-279.

> 2. In the response, the authors mention "In particular our current framework does not immediately have options for including covariates in the multivariate analysis, so correcting for confounders such as population structure should be done on the first set of univariate analyses. We have added specific mention of this to the Discussion." In the Discussion, I found this text: "in many cases summary data remain much easier to obtain and work with; there are big practical advantages as well to modular pipelines that first compute summary data and then use these as inputs to subsequent (more sophisticated) analyses. For example, the multivariate analyses we present here are simplified by assuming that the summary data were computed while adequately adjusting for population stratification." I may have missed other relevant text but the above quoted text from Discussion does not seem to emphasize/convey the message that any necessary covariate adjustment *has* to be done before obtaining the single-trait GWAS summary statistics, and cannot be done at the multivariate analysis step. So, if one has access to GWAS summary data from a study that did not adjust for population stratification, one cannot expect it to be taken care of by the multivariate analysis.

We agree with the reviewer and have made it more clear that correction for covariates **must** be done prior to running `bmass` (edits on lines 267-272).

> 3. In the response, the authors mention "Under the scenario where a consortium finds that running a multivariate analysis does not indicate any new SNPs as being genome-wide significant, then we would not criticize them for choosing to focus on univariate analyses. We have added a discussion of this to the manuscript." I have probably missed but I couldn't find this discussion in the manuscript.

We considered the addition of lines 248-253 in the third paragraph of 'Limitations' to be the new discussion referenced here.

> 4. It is not clear if the BFav approach is only applicable to continuous traits (if I am not mistaken, the authors have analyzed continuous traits only in this manuscript). If so, it is worth emphasizing.

It is also possible to analyze binary traits with our approach. The justification comes from Wakefield (2009), who points out that the likelihood for the summary data from binary traits is approximately normal. We have added a reference to this on lines 81-85.

Response to Editor

> -We notice that you used "unpublished" 1 time in the manuscript, on page 27. PLOS Genetics recently updated its data access policy and now requires that all data be either published with the manuscript or made available in a publically accessible database. We ask that you either amend the supplementary material to include the referenced data or remove as many of the references as possible. Please include an explanation of how you have met this request (i.e. whether you have simply removed the instances because they are not integral to the manuscript, or whether you have provided further supporting information files) at the end of your response to reviewers. Please note that we will not be able to pass your manuscript through our technical checks unless this is accompanied by this explanation.

Our use of "unpublished" on page 27, line 402 is in reference to the previous studies we collected data from. We are classifying some of our current results as possibly having been 'unpublished' from the previous studies (eg results that possibly should have been published by the previous studies but for some unknown reasons were not) – so we are not actually discussing results that we, ourselves, are

keeping unpublished. Our results are still shared. We hope this clarifies our use of "unpublished".

References

Wakefield, J. (2009). Bayes factors for genome-wide association studies: comparison with p-values. *Genet Epidemiol*, 33(1):79–86.
